# Clinicopathological Characteristics of Upper Tract Urothelial Cancer With Loss of Immunohistochemical Expression of Mismatch Repair Proteins

**DOI:** 10.1111/iju.70146

**Published:** 2025-06-09

**Authors:** Go Kobayashi, Tetsutaro Hayashi, Yohei Sekino, Kenichiro Ikeda, Hikaru Nakahara, Kohei Kobatake, Keisuke Goto, Daiki Taniyama, Kazuya Kuraoka, Shintaro Akabane, Hiroaki Niitsu, Takao Hinoi, Kazuhiro Sentani, Nobuyuki Hinata

**Affiliations:** ^1^ Laboratory of Molecular Pathology, Department of Molecular Biosciences Radiation Effects Research Foundation Hiroshima Japan; ^2^ Department of Molecular Pathology, Graduate School of Biomedical and Health Sciences Hiroshima University Hiroshima Japan; ^3^ Department of Urology, Graduate School of Biomedical and Health Sciences Hiroshima University Hiroshima Japan; ^4^ Department of Clinical and Molecular Genetics Hiroshima University Hospital Hiroshima Japan; ^5^ Department of Diagnostic Pathology, Kure Medical Center and Chugoku Cancer Center National Hospital Organization Kure Japan; ^6^ Department of Gastroenterological and Transplant Surgery, Graduate School of Biomedical and Health Sciences Hiroshima University Hiroshima Japan

**Keywords:** clinicopathological significance, immunohistochemistry, lynch syndrome, mismatch repair, upper tract urothelial carcinoma

## Abstract

**Objectives:**

Lynch syndrome (LS) is an inherited cancer predisposition caused by germline mutations in DNA mismatch repair (MMR) genes. Upper tract urothelial carcinoma (UTUC) is the third most common cancer associated with LS. In this study, we examined MMR protein expression in UTUC using immunohistochemistry to clarify the clinicopathological characteristics and prognostic significance of LS‐associated UTUC.

**Methods:**

We analyzed the expression of MLH1, MSH2, MSH6, and PMS2 by immunohistochemistry in 118 cases of UTUC treated with radical nephroureterectomy. MMR deficiency was defined as tumors exhibiting less than 5% MMR protein expression. We conducted further investigations using public databases.

**Results:**

MMR deficiency was identified in 15 (13%) of the 118 UTUC cases. These cases were associated with younger age, papillary morphology, low grade, low stage, low neutrophil‐to‐lymphocyte ratio, high levels of CD8‐positive tumor‐infiltrating lymphocytes, and favorable prognosis. Similar findings were observed through in silico analysis. Public datasets revealed that tumor mutational burden in UTUC was significantly higher in MMR‐mutated cases compared to MMR‐normal cases. A waterfall plot showed a high frequency of FGFR3 mutation in MMR‐mutated cases in UTUC. Bioinformatics analysis using RNA‐Seq datasets showed that MMR‐mutated UTUC was associated with enriched gene sets for MYC targets v1 and oxidative phosphorylation. Furthermore, gene expression levels of GALNT12 and FRMD3 emerged as potential predictors of MMR mutation in UTUC.

**Conclusions:**

These findings highlight the clinical value of evaluating MMR protein expression by immunohistochemistry, which could inform treatment strategies and surveillance protocols for UTUC patients.

## 1 Introduction

Urothelial carcinoma (UC) is a common malignant disease. Most UC cases are of urinary bladder cancer (BLCA). Upper tract UC (UTUC) is relatively rare, accounting for approximately 5%–10% of all urothelial tumors [1]. Recent genomic studies revealed that UC can be classified into molecular subtypes, enabling precision medicine [2]. Moreover, examination of microsatellite instability (MSI) has become an increasingly important biomarker in UTUC [3]. MSI is described in many cancers, and for advanced solid MSI tumors, targeted therapy with immune checkpoint inhibitors is available [3]. MSI is also well known to be associated with the presence of Lynch syndrome (LS). LS is an inherited cancer caused by germline mutations in DNA mismatch repair (MMR) genes including MLH1, MSH2, MSH6, and PMS2, and UTUC is reported as the third most frequent cancer in LS [4].

Evaluation of MMR proteins by immunohistochemistry (IHC) and MSI testing is a widely used screening method [5]. Thus, MMR deficiency identified by IHC is useful to predict clinical benefits of immune checkpoint blockade with anti‐PD‐1 antibody therapy. Recent studies on UTUC have reported a prevalence of MMR deficiency ranging from 3% to 19% [3, 6, 7, 8]. Clinically, MMR‐deficient UTUC tends to present at a younger age and an early stage and is histologically classified as a low‐grade tumor with distinct pathological features [8, 9, 10]. However, to our best knowledge, detailed information on the clinicopathological characteristics and prognostic significance of MMR deficiency in UTUC remains limited. Furthermore, key signaling pathways and molecular mechanisms underlying MMR‐deficient UTUC are largely unknown.

To assess the prevalence and clinicopathological characteristics of UTUC with loss of MMR genes, we investigated the expression of MMR proteins immunohistochemically and compared clinicopathological features between patients with UTUC with and without MMR deficiency. Additionally, we conducted further investigations using public databases. By utilizing RNA‐seq data from UTUC, we explored differences in gene functions between MMR‐mutated and MMR‐normal cases through a bioinformatics approach.

## 2 Materials and Methods

### 2.1 Tissue Samples

We retrospectively reviewed medical records of 118 patients (mean age 71.4 ± 10.2 years, 75% male) who underwent radical nephroureterectomy for unilateral UTUC at Hiroshima University Hospital (April 1999–December 2015). Patients receiving neoadjuvant chemotherapy were excluded. Patients who received adjuvant immunotherapy were also not included in this study. Cancer history was available for 109 patients, whereas it remained unclear for nine patients. Additionally, C‐reactive protein (CRP) levels were not confirmed for five patients. Pathology specimens were re‐evaluated using the 8th AJCC/UICC TNM classification (2017) and the 1973 WHO ISUP grading system. Among 118 UTUC cases, during follow‐up, disease progression occurred in 29 patients (25%), with 18 (15%) dying from the disease. Study endpoints were CSS and PFS, with progression defined as lymph node relapse or distant metastasis (excluding bladder cancer recurrence or contralateral UTUC). The median follow‐up period was 63.0 months (IQR: 25,2–114.8 months) for CSS and 53.2 months (IQR: 13.6–93.3 months) for PFS. Follow‐up included urine analysis and chest‐abdomen‐pelvis CT every 3–6 months for at least 5 years, per physician preference. The final follow‐up date was March 25, 2025.

### 2.2 Immunohistochemistry

IHC was performed on 1–2 representative tumor blocks using 3–4 μm slides, as previously reported [11]. Antibodies for MLH1 (1:100, ES05, IR079), MSH2 (1:100, FE11, IR085), MSH6 (1:100, EP49, IR086), and PMS2 (1:50, EP51, IR087) were used for MMR assessment. Positive control slides from LS‐related colorectal or gastric cancer (MLH1/PMS2 loss) ensured staining quality (Figure S1). MMR deficiency was defined as < 5% staining for at least one MMR protein. Evaluation of GATA3, CK5/6, TP53, CD44v9, PD‐L1, and CD8 was described previously [12, 13].

### 2.3 In Silico Analysis

The dataset from the study by Fujii et al., which included the clinicopathological characteristics and RNA‐seq data of UTUC patients, were obtained [2] for conduction of survival and bioinformatics analyses. Additional public datasets, including TCGA (for UTUC and BLCA), UTUC Eur Urol 2015, and the AACR Project GENIE (for UTUC and BLCA), were also utilized, with corresponding references provided in the Data S2. The TCGA data were accessed via cBioPortal (https://www.cbioportal.org). The median follow‐up was 40.7 months (IQR: 23.6–80.5) for CSS and 33.8 months (IQR: 15.0–77.2) for PFS in Fujii et al., 33.1 months (IQR: 15.7–76.6) for OS in the UTUC Eur Urol 2015 cohort, and 17.6 months (IQR: 10.8–31.4) in the BLCA TCGA cohort. In the TCGA UTUC dataset, the histological grade information was unavailable for 3 cases. Details of bioinformatics analysis are provided in Data S3.

### 2.4 Statistical Analysis

All statistical analyses were performed using SPSS (SPSS Inc., Chicago, IL, USA). Fisher's exact test assessed correlations between clinicopathological parameters and MMR deficiency. Kaplan–Meier curves with log‐rank tests compared survival between MMR‐deficient and MMR‐normal groups. Mann–Whitney U test evaluated MMR mutation counts. Spearman's rank correlation test assessed correlations between GALNT12 and FRMD3 expression levels. LASSO Cox regression identified key MMR mutation predictors. Statistical significance was set at *p* < 0.05. Waterfall plot, UMAP, ROC, and SVM analyses were performed using Python 3.0 on Jupyter Notebook. The waterfall plot codes are in Data S1, and others were described previously [14].

## 3 Results

### 3.1 Relationship Between MMR Deficiency, Clinicopathological Parameters, and Prognosis in Our Cohort

**FIGURE 1:**
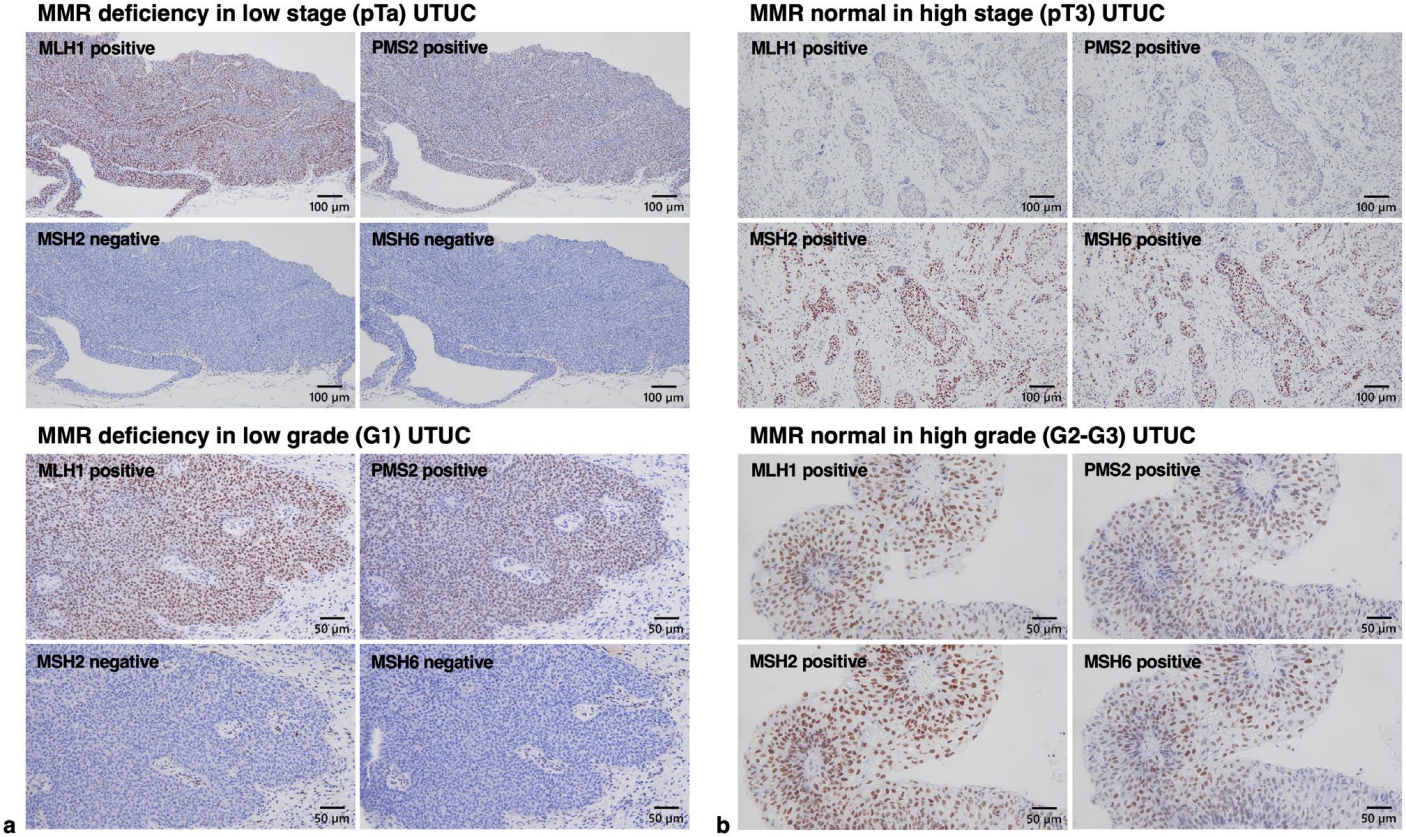
Representative immunohistochemical images of mismatch repair (MMR) deficiency and MMR‐normal cases in upper tract urothelial carcinoma (UTUC). (a) Representative images of MMR‐deficient UTUC in low‐stage or low‐grade cases, showing complete loss of MSH2 and MSH6 expression. (b) Representative images of MMR‐normal UTUC in high‐stage or high‐grade cases. Scale bars indicate 100 μm or 50 μm.

**FIGURE 2:**
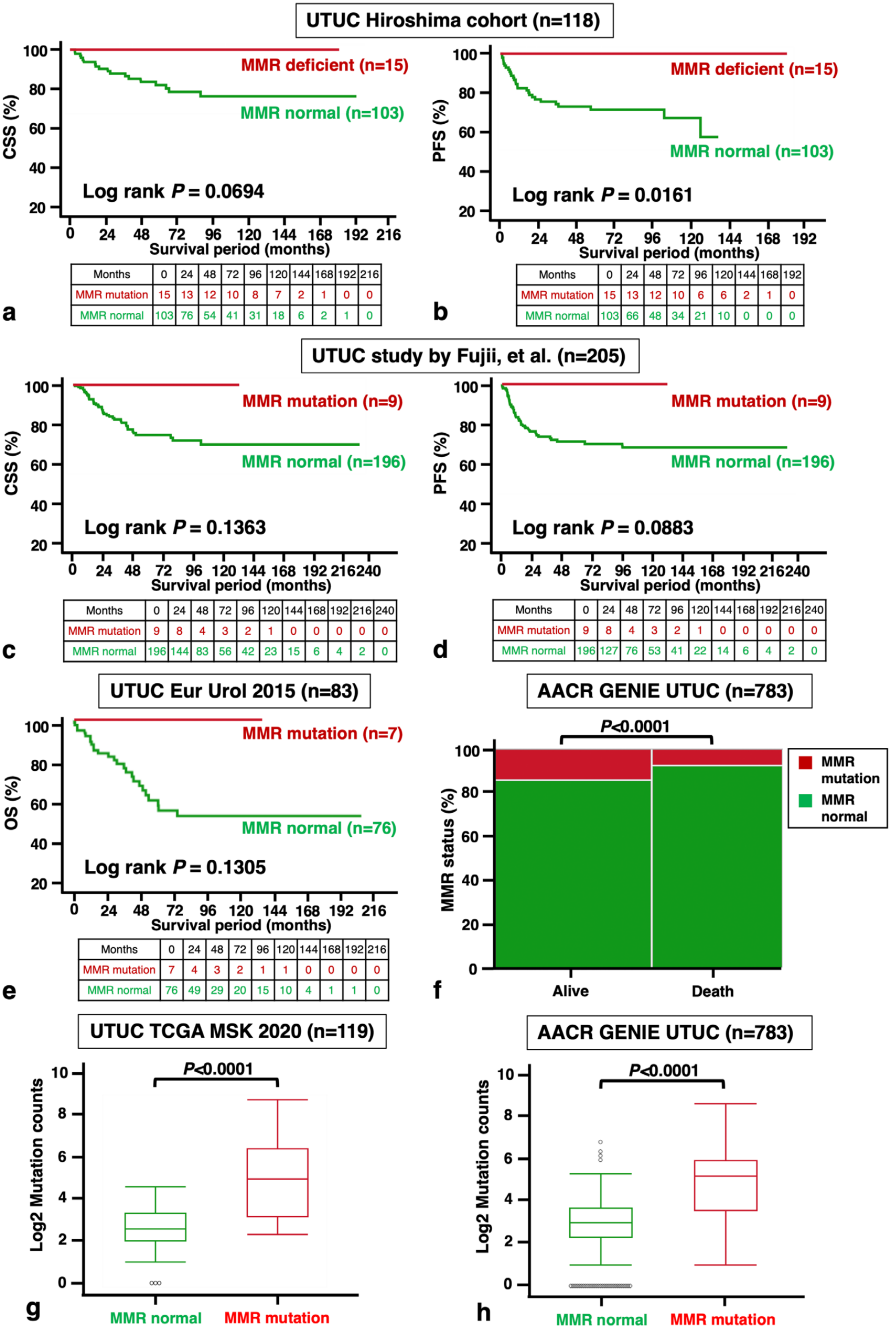
Survival analysis. Kaplan–Meier plots illustrating (a) cancer‐specific survival (CSS) and (b) progression‐free survival (PFS) of upper tract urothelial cancer (UTUC) patients stratified by tumor mismatch repair (MMR) expression in the Hiroshima cohort; (c) CSS and (d) PFS from the UTUC study by Fujii et al. [13]; and (e) overall survival (OS) from the TCGA UTUC study (Eur Urol, 2015). (f) Proportion of MMR mutation versus MMR normal cases among surviving and deceased patients in the AACR GENIE UTUC study. Comparison of tumor mutation burden (TMB) between MMR‐mutated and MMR‐normal cases in (g) the TCGA UTUC study (MSK, 2020) and (h) the AACR GENIE UTUC study. Statistical significance was assessed using the Mann–Whitney *U* test.

Immunohistochemical staining was performed on 118 UTUC samples, with representative images of MMR deficiency shown in Figure 1. MMR deficiency was detected in 15 cases (13%) (Table 1) and was significantly associated with younger age (*p* = 0.0256), G1/G2 grade (*p* = 0.0180), low pT stage (*p* = 0.0117), and absence of carcinoma in situ (CIS) (*p* = 0.0128) (Table 2). Additionally, MMR‐deficient cases had a lower neutrophil‐lymphocyte ratio (NLR) (*p* = 0.0377) (Table 3). We analyzed the association between MMR deficiency and cancer‐related markers, including GATA3, CK5/6, TP53, Ki‐67, PD‐L1, and CD8. MMR deficiency correlated significantly with CD8 expression in TILs (*p* = 0.0141) (Table 4), and CD8 positivity in TILs was linked to PD‐L1 positivity (Table S1). Kaplan–Meier analysis showed that patients with MMR deficiency showed better CSS compared with the others, although the difference did not reach statistical significance (*p* = 0.0694, Figure 2a). MMR deficiency was significantly linked to increased PFS (*p* = 0.0161, Figure 2b).

**TABLE 1:** Distribution of MMR mutation in UTUC Hiroshima cohort and other public dataset.

UTUC Hiroshima cohort	UTUC by Fuji et al.	UTUC TCGA MSK 2020	BLCA TCGA
MSH2/MSH6 (*n* = 3)	MLH1 (*n* = 1)	MLH1/MSH2/MSH6 (*n* = 1)	MLH1/PMS2 (*n* = 1)
MLH1/PMS2 (*n* = 2)	MSH2 (*n* = 3)	MSH2/MSH6 (*n* = 1)	MSH2/MSH6 (*n* = 1)
MLH1/PMS2/MSH6 (*n* = 1)	MSH6 (*n* = 5)	MLH1 (*n* = 4)	MLH1/MSH6 (*n* = 1)
MLH1/MLH6 (*n* = 1)		MSH2 (*n* = 6)	MLH1 (*n* = 7)
MSH6 (*n* = 8)		MSH6 (*n* = 2)	MSH2 (*n* = 12)
		PMS2 (*n* = 1)	MSH6 (*n* = 9)
			PMS2 (*n* = 9)
15 (13%) of 118 cases	9 (4%) of 205 cases	15 (13%) of 119 cases	40 (10%) of 412 cases

Abbreviations: BLCA, bladder cancer; TCGA, The Cancer Genome Atlas; UTUC, upper tract urothelial carcinoma.

**TABLE 2:** Relationship between MMR deficiency and clinicopathological characteristics in 118 UTUC cases in Hiroshima cohort.

Factors	MMR deficiency (*n* = 15)	MMR normal (*n* = 103)	*p*
Gender			
Male (*n* = 88)	11 (13%)	77 (87%)	> 0.9999
Female (*n* = 30)	4 (13%)	26 (87%)	
Age (Ave ± SD)	65.7 ± 9.6	71.8 ± 10.0	**0.0256**
BMI (Ave ± SD)	24.2 ± 3.1	22.5 ± 3.1	0.0943
Side			
Right (*n* = 57)	7 (12%)	50 (88%)	0.8919
Left (*n* = 61)	8 (13%)	53 (87%)	
History of cancer			
Positive (*n* = 41)	9 (22%)	32 (78%)	0.083
Negative (*n* = 68)	6 (9%)	62 (91%)	
BLCA			
Positive (*n* = 40)	6 (15%)	34 (85%)	0.3103
Negative (*n* = 78)	9 (12%)	69 (88%)	
Tumor location			
Renal (*n* = 59)	11 (19%)	48 (81%)	0.0993
Ureter (*n* = 54)	4 (7%)	50 (93%)	
Renal/Ureter (*n* = 5)	0 (0%)	5 (100%)	
Tumor morphology			
Papillary (*n* = 69)	12 (17%)	57 (83%)	0.0702
Nodular/Flat (*n* = 49)	3 (6%)	46 (94%)	
Histology			
Pure UC (*n* = 108)	14 (13%)	94 (87%)	> 0.9999
Variants (*n* = 10)	1 (10%)	9 (90%)	
With CIS			
Positive (*n* = 49)	3 (6%)	46 (94%)	**0.0128**
Negative (*n* = 69)	12 (17%)	57 (83%)	
Histological grade			
G1/2 (*n* = 77)	14 (18%)	63 (82%)	**0.0180**
G3 (*n* = 41)	1 (3%)	40 (97%)	
pT stage			
pTis/a/1 (*n* = 57)	12 (21%)	45 (79%)	**0.0117**
pT2/3/4 (*n* = 61)	3 (5%)	58 (95%)	
pTis/a/1/2 (*n* = 65)	12 (18%)	53 (82%)	**0.0311**
pT3/4 (*n* = 53)	3 (6%)	50 (94%)	
pN stage			
pN0/X (*n* = 113)	15 (13%)	98 (87%)	> 0.9999
pN1/2 (*n* = 5)	0	5 (100%)	

*Note:* *p* Values were calculated with Fisher's exact test. Bold values show the statistical significance. BLCA refers to both concomitant and prior history of bladder cancer.

Abbreviations: Ave, average; BLCA, bladder cancer; CIS, carcinoma in situ; MMR, mismatch repair; SD, standard deviation.

**TABLE 3:** Relationship between MMR deficiency and blood test results in 118 UTUC cases in Hiroshima cohort.

Factors	MMR deficiency	MMR normal	*p*
White blood cells (Ave ± SD)	6523 ± 2182	6471 ± 2080	0.9193
Neutrophils (Ave ± SD)	3833 ± 1272	4035 ± 1620	0.8181
Lymphocytes (Ave ± SD)	2191 ± 946	1760 ± 762	0.1028
NLR (Ave ± SD)	1.88 ± 0.62	2.55 ± 1.12	**0.0377**
Hemoglobin (Ave ± SD)	13.1 ± 1.22	12.9 ± 1.90	0.7349
Platelet (Ave ± SD)	20.95 ± 4.871	22.29 ± 7.457	0.5763
Albumin (Ave ± SD)	4.15 ± 0.42	4.17 ± 0.38	0.8151
Creatinine (Ave ± SD)	1.03 ± 0.62	1.20 ± 1.13	0.1973
CRP < 0.3	11 (14%)	70 (86%)	0.7537
CRP 0.3 ≦	3 (9%)	29 (91%)	

*Note:* *p* Values were calculated with Fisher's exact test. Bold values show the statistical significance.

Abbreviations: Ave, average; CRP, C‐reactive protein; MMR, mismatch repair; NLR, Neutrophil‐Lymphocyte Ratio; SD, standard deviation.

**TABLE 4:** Relationship between MMR deficiency and various cancer‐related molecules in UTUC in Hiroshima cohort.

IHC markers	MMR deficiency	MMR normal	*p*
GATA3			
Positive	12 (12%)	92 (88%)	0.3845
Negative	3 (21%)	11 (79%)	
Cytokeratin 5/6			
Positive	5 (20%)	20 (80%)	0.3067
Negative	10 (11%)	83 (89%)	
TP53			
Positive	2 (6%)	33 (94%)	0.2254
Negative	13 (17%)	70 (83%)	
CD44v9			
Positive	1 (4%)	27 (96%)	0.1158
Negative	14 (16%)	76 (84%)	
PD‐L1 in TCs			
Positive	1 (6%)	16 (94%)	0.6932
Negative	14 (14%)	87 (86%)	
PD‐L1 in TILs			
Positive	3 (8%)	34 (92%)	0.3846
Negative	12 (15%)	69 (85%)	
CD8 in TILs			
Positive	9 (25%)	27 (75%)	**0.0141**
Negative	6 (7%)	76 (93%)	

*Note:* *p*‐Values were calculated with Fisher's exact test. Bold values show the statistical significance.

Abbreviations: CK 5/6, cytokeratin 5/6; GATA3, GATA binding protein 3; MMR, mismatch repair; PD‐L1, programmed death ligand 1; TCs, tumor cells; TILs, tumor‐infiltrating lymphocyte.

### 3.2 Investigation of MMR Mutation in UTUC Using Public Databases

Using data from Fujii et al. [2], we analyzed the relationship between MMR mutations and clinicopathological parameters. MMR mutations were detected in 9 of 205 UTUC cases (4%) and were significantly associated with the hypermutated (*p* < 0.0001) and Cluster 1 subtypes (*p* = 0.0182) (Table 5). Although not statistically significant, MMR‐mutated cases showed a trend toward better CSS and PFS (Figure 2c,d). In the TCGA UTUC dataset (MSK, 2020), MMR mutations were found in 15 of 119 cases (13%) and were significantly associated with younger age (Table 5). In the Eur Urol UTUC dataset (2015), MMR‐mutated cases had a more favorable prognosis (Figure 2e), and their proportion was significantly higher among surviving patients in the AACR GENIE UTUC dataset (Figure 2f). TMB was also significantly elevated in MMR‐mutated cases across the TCGA UTUC and AACR GENIE UTUC datasets (Figure 2g,h). In the TCGA BLCA dataset, MMR mutation distribution is shown in Table 1. MMR mutations were significantly linked to favorable prognosis (*p* = 0.0159, Figure S2a), higher survival rates in the AACR GENIE BLCA dataset (Figure S2b), and increased TMB in both TCGA BLCA and AACR GENIE BLCA datasets (Figure S2c,d).

**TABLE 5:** Relationship between MMR mutation and clinicopathological characteristics in UTUC public datasets.

UTUC study by Fuji et al. (*n* = 205)	MMR mutation (*n* = 9)	MMR normal (*n* = 196)	*p*
Gender			
Male (*n* = 141)	5 (4%)	136 (96%)	0.3812
Female (*n* = 64)	4 (6%)	60 (94%)	
Age (Ave ± SD)	68.9 ± 5.8	70.9 ± 9.6	0.6398
Tumor morphology			
Papillary (*n* = 175)	9 (5%)	166 (95%)	0.2040
Nodular/Flat (*n* = 30)	0	30 (100%)	
Histological grade			
Low grade (*n* = 90)	4 (4%)	86 (96%)	0.9733
High grade (*n* = 115)	5 (4%)	110 (96%)	
pT stage			
pTis/a/1 (*n* = 100)	6 (6%)	94 (94%)	0.2723
pT2/3/4 (*n* = 105)	3 (3%)	102 (97%)	
Mutational subtype			
FGFR3 (*n* = 71)	0	71 (100%)	**< 0.0001**
Hypermutated (*n* = 14)	9 (64%)	5 (36%)	
RAS (*n* = 31)	0	31 (100%)	
TP53 (*n* = 76)	0	76 (100%)	
Triple‐negative (*n* = 13)	0	13 (100%)	
CNA subtype			
Cluster 1 (*n* = 125)	9 (7%)	116 (93%)	**0.0182**
Cluster 2 (*n* = 74)	0	74 (100%)	

TCGA UTUC (*n* = 119)	MMR mutation (*n* = 15)	MMR normal (*n* = 104)	*p*
Gender			
Male (*n* = 78)	10 (13%)	68 (87%)	> 0.9999
Female (*n* = 41)	5 (12%)	36 (88%)	
Age (Ave ± SD)	62.3 ± 11.4	69.3 ± 9.9	**0.0316**
Histological grade			
Low grade (*n* = 23)	2 (9%)	21 (91%)	0.732
High grade (*n* = 93)	13 (14%)	80 (86%)	
pT stage			
pTa/is/1 (*n* = 62)	7 (11%)	55 (89%)	0.784
pT2/3/4 (*n* = 57)	8 (14%)	49 (86%)	

*Note:* *p* Values were calculated with Fisher's exact test. Bold values show the statistical significance.

Abbreviations: Ave, average; CAN, copy number alterations; FGFR3, fibroblast growth factor receptor3; MMR, mismatch repair; SD, standard deviation; TCGA, The Cancer Genome Atlas; UTUC, upper tract urothelial carcinoma.

**FIGURE 3:**
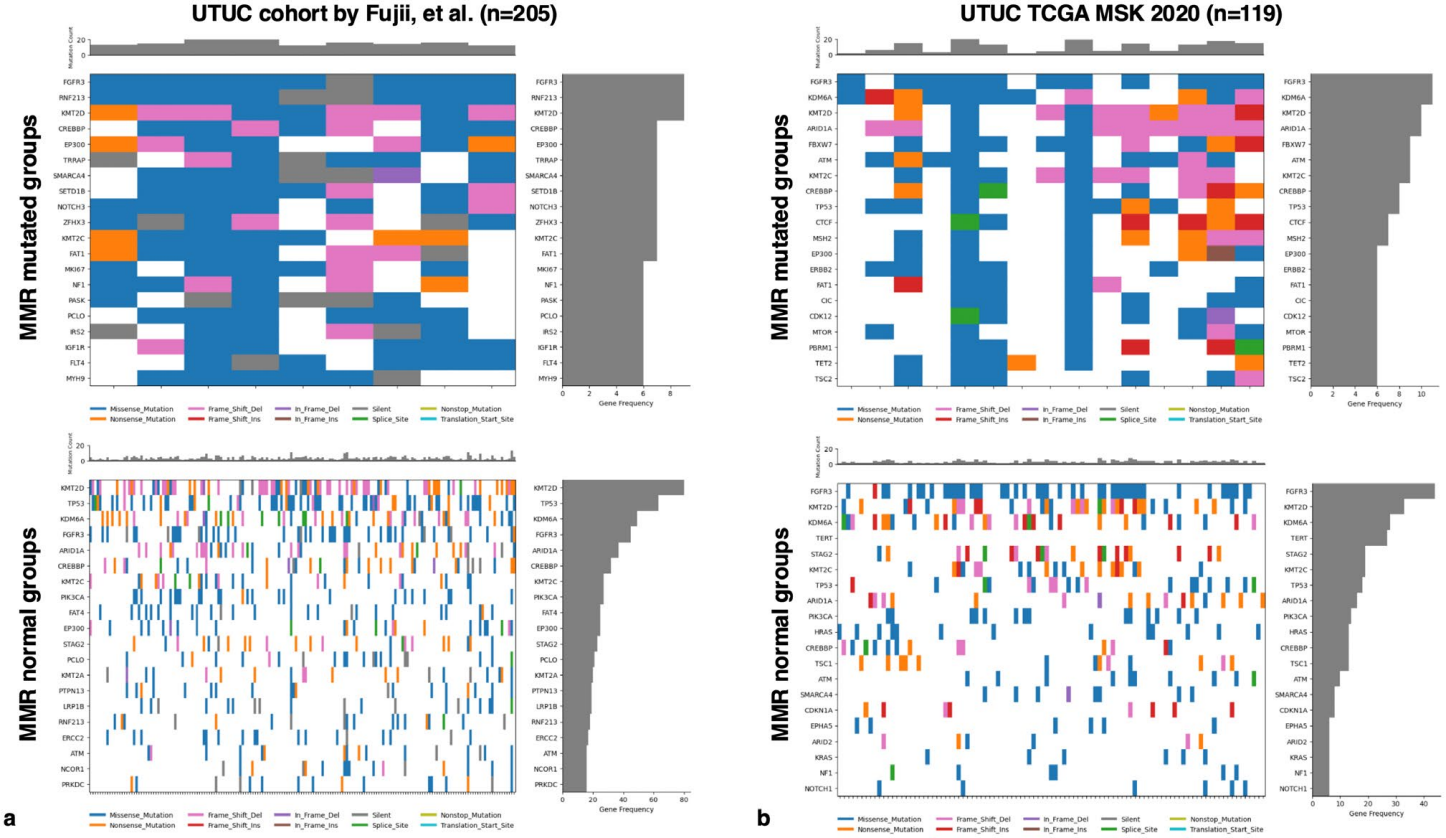
Waterfall plot depicting the distribution of mutations in upper tract urothelial carcinoma (UTUC). The upper panel shows the mutation frequency for each tumor sample, whereas the right panel displays the frequency of gene mutations. The main tile plot illustrates the types of mutations in each UTUC case. The top 20 genes are presented in descending order based on mutation frequency. Waterfall plots of mismatch repair (MMR)‐mutated and MMR‐normal cases are shown for (a) the UTUC study by Fujii et al. and (b) the TCGA UTUC study (MSK, 2020).

Somatic mutation rates were compared in the Fujii et al. [2] and TCGA UTUC datasets. In MMR‐mutated cases, the most frequently mutated genes in both cohorts included FGFR3, KMT2D, CREBBP, EP300, and FAT1 (Figure 3). KMT2D, KDM6A, FGFR3, TP53, ARID1A, and KMT2C were frequently mutated in MMR‐normal cases (Figure 3).

### 3.3 Bioinformatics Analysis of MMR Mutation in UTUC Using RNA‐Seq Dataset

**FIGURE 4:**
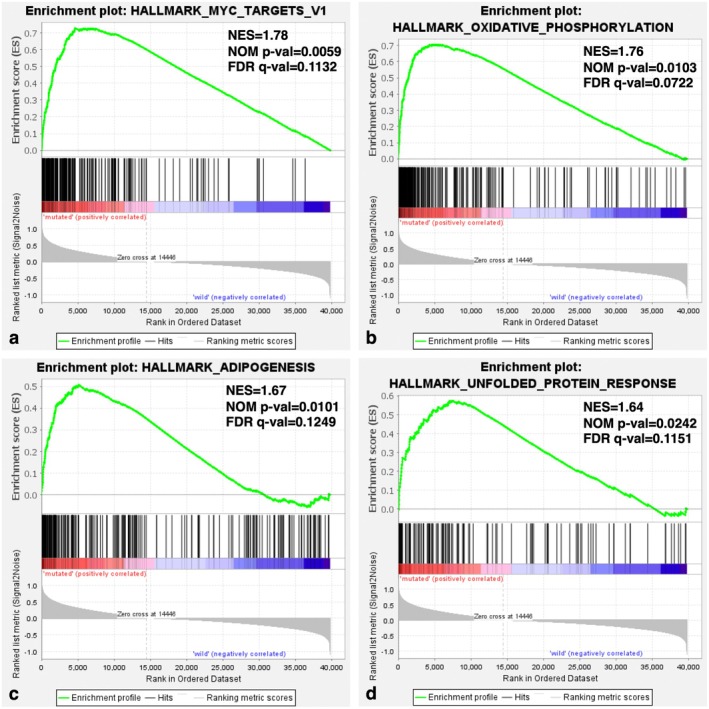
Gene set enrichment analysis (GSEA) of mismatch repair (MMR)‐mutated and ‐normal groups. (a) Enrichment plot of HALLMARK_MYC_TARGETS_V1. (b) Enrichment plot of HALLMARK_OXIDATIVE_PHOSPHORYLATION. (c) Enrichment plot of HALLMARK_ADIPOGENESIS. (d) Enrichment plot of HALLMARK_UNFOLDED_PROTEIN_RESPONSE.

**FIGURE 5:**
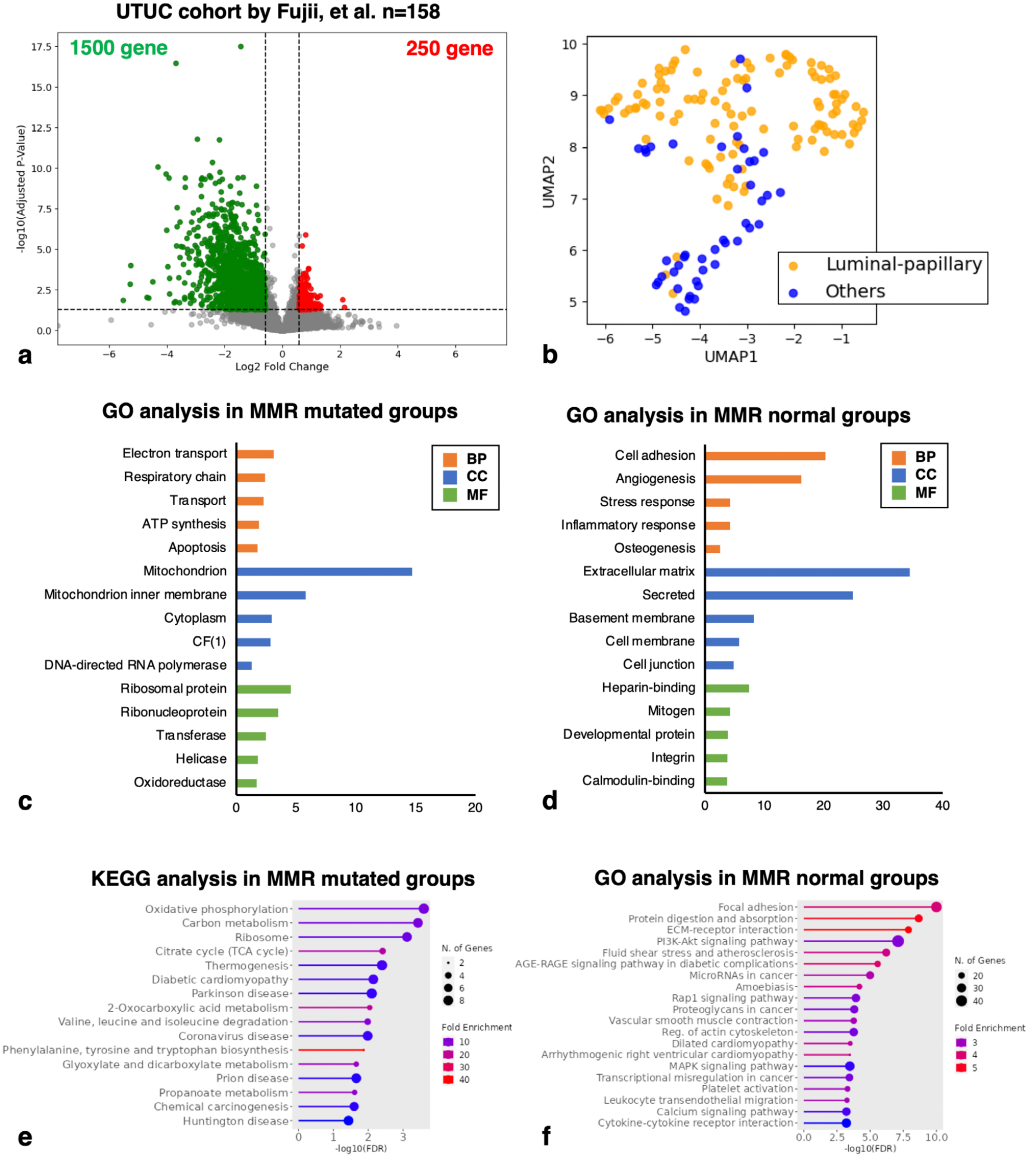
Identification and functional enrichment analysis of differentially expressed genes (DEGs) between mismatch repair (MMR)‐mutated and MMR‐normal groups in upper tract urothelial cancer (UTUC). (a) Volcano plot of DEGs. Each point represents an individual gene. The *X*‐axis shows the log_2_ fold change in gene expression (MMR mutated/normal), and the *Y*‐axis shows the ‐log10 adjusted *p*‐value. The vertical dotted lines indicate a 1.5‐fold change threshold, and the horizontal dotted line denotes an adjusted *p*‐value of 0.05. Genes in red are upregulated in MMR‐mutated UTUC, whereas those in green are downregulated. (b) Uniform Manifold Approximation and Projection (UMAP) distribution plots of UTUC cases based on DEGs. (c, d) Gene Ontology (GO) enrichment analysis of upregulated genes in (c) MMR‐mutated and (d) MMR‐normal groups. (e, f) Kyoto Encyclopedia of Genes and Genomes (KEGG) enrichment analysis of upregulated genes in (e) MMR‐mutated and (f) MMR‐normal groups.

The 158 UTUC samples from Fujii et al. [2] were classified into MMR‐mutated and MMR‐normal groups. GSEA identified significant associations in the MMR‐mutated group with MYC targets v1, OXPHOS, adipogenesis, and UPR (Figure 4). A volcano plot revealed 250 upregulated genes in the MMR‐mutated group and 1500 in the MMR‐normal group (fold change > 1.5, *p* adj. < 0.05; Figure 5a). UMAP of 1750 DEGs showed a tendency to distinguish luminal papillary subtypes (Figure 5b).

GO analysis indicated that MMR‐mutated upregulated genes were enriched in mitochondrial and ribosomal processes, while MMR‐normal upregulated genes were linked to ECM, secretion, adhesion, and angiogenesis (Figure 5c,d). KEGG analysis suggested MMR‐mutated genes were involved in OXPHOS, carbon metabolism, ribosome biogenesis, and the citrate cycle, whereas MMR‐normal genes were associated with FA, ECM‐receptor interaction, protein digestion, and PI3K‐Akt signaling (Figure 5e,f).

### 3.4 Prediction of MMR Mutation in UTUC Using RNA‐Seq Dataset

**FIGURE 6:**
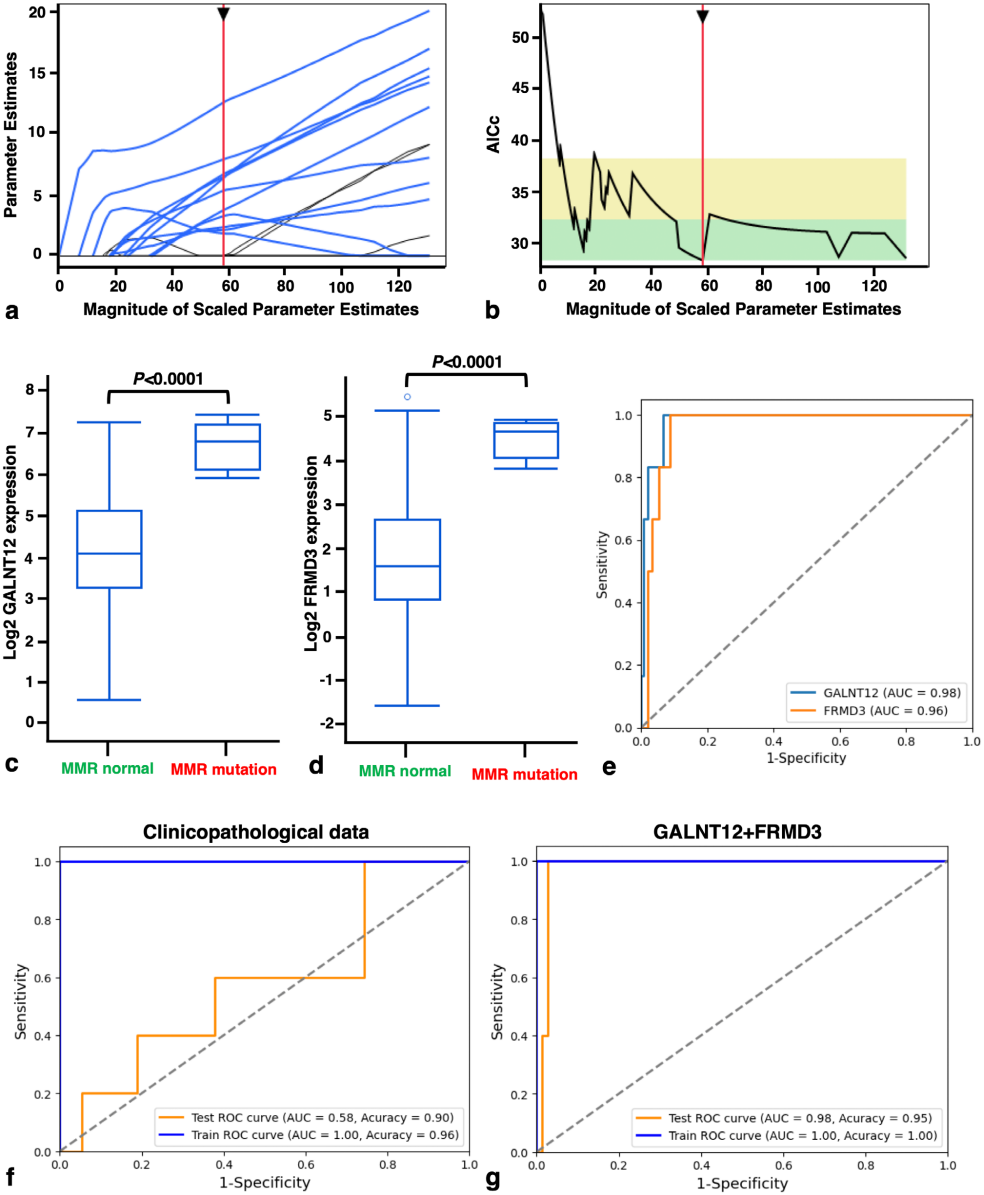
Identification of potential genes for predicting mismatch repair (MMR) mutation. (a) Least absolute shrinkage and selection operator (LASSO) profiles showing parameter estimates for selected genes. (b) Tuning parameter selection in LASSO, with the minimum Akaike information criterion (corrected) indicating optimal model performance. (c, d) Comparison of GALNT12 and FRMD3 gene expression levels between MMR‐mutated and MMR‐normal groups. Statistical significance was assessed using the Mann–Whitney *U* test. (e) Receiver operating characteristic (ROC) curve analysis for predicting MMR mutation using GALNT12 and FRMD3 gene expression. (f) ROC curves comparing the classification of MMR mutation based on the clinicopathological dataset. (g, e) ROC curves for classification of MMR mutation using a support vector machine (SVM) algorithm based on (g) the clinicopathological dataset and (e) GALNT12 and FRMD3 gene expression data.

We explored gene‐based prediction of MMR mutation in UTUC. Lasso regression of 250 upregulated genes in the MMR‐mutated group identified 11 key predictors (Figure 6a,b; Table S2), with GALNT12 and FRMD3 standing out due to significant *p*‐values and large regression coefficients. Their RNA‐seq expression was significantly higher in MMR‐mutated UTUC (Figure 6c,d). ROC analysis confirmed their strong predictive potential with AUCs of 0.98 and 0.96, respectively (Figure 6e). An SVM model trained on clinicopathological features (age, sex, tumor morphology, grade, pT stage, mutational subtype, CNA subtype) yielded an AUC of 0.58 and 90% accuracy. Incorporating GALNT12 and FRMD3 expression markedly improved performance, achieving an AUC of 0.98 and 95% accuracy (Figure 6f,g).

Additionally, we analyzed the prognostic relevance of GALNT12 and FRMD3 in 158 UTUC cases [2]. Before performing the prognostic analysis, we confirmed a significant correlation between GALNT12 and FRMD3 expression (Figure S3a). Interestingly, FRMD3 expression levels were significantly higher among patients who survived or did not experience disease progression (Figure S3b,c). Survival curves were generated based on median cutoff values. Although GALNT12 expression was not significantly associated with prognosis, FRMD3 expression showed a significant correlation with favorable CSS and PFS (Figure S3d–g).

## 4 Discussion

We identified a prevalence of 13% of UTUC cases exhibiting MMR deficiency and potential LS. Compared to non‐MMR‐deficient UTUC, patients with MMR‐deficient UTUC were associated with younger age, low histological grade, low pathological T stage, and absence of concomitant CIS. At our facility, all urothelial tissues in UTUC and BLCA specimens are thoroughly examined under pathologist supervision, which may lead to a higher detection rate of concomitant CIS than at other institutions. Moreover, patients with MMR‐deficient UTUC had a better prognosis. Similar findings were confirmed through in silico analysis. Furthermore, several public datasets showed that TMB was upregulated in MMR‐mutated cases in both UTUC and BLCA cohorts. Indeed, MMR‐mutated cases were significantly associated with the hypermutated subtype in the UTUC cohort of Fuji et al. [2]. Depending on the molecular subtype, clinical behaviors and responses to chemotherapy are distinct [2], suggesting that determination of MMR status may be helpful for clinical decision‐making.

In many tumors, high TMB and MSI are associated with a better outcome and improved response to adjuvant chemotherapy and immunotherapy regimes [5]. MSI is closely associated with MMR deficiency. Thus, confirming MMR status in UTUC is considered crucial for effective systemic treatment. Additionally, our study found that MMR‐deficient UTUC is associated with less pathologically aggressive features, consistent with previous findings [8, 10], and moreover, that MMR deficiency was associated with low NLR. A preoperative low NLR is considered a good prognostic indicator in UTUC [15]. Additionally, MMR‐mutated cases were strongly associated with the hypermutated subtype, which is characterized by a high frequency of FGFR3 mutations and a favorable prognosis in UTUC [2]. Indeed, a waterfall plot showed a high frequency of FGFR3 mutation in MMR‐mutated cases in UTUC. Generally, FGFR3 alterations are well documented in UTUC and are typically associated with low T grade, non‐muscle‐invasive tumors and a favorable prognosis, further supporting our findings. This may partly explain the less aggressive features seen in MMR‐deficient cases. Thus, patients with MMR deficiency are considered to have low malignant potential and may not require additional treatment. Recently, FGFR3 inhibitors, erdafitinib, have been approved by the FDA as the treatment for certain patients with UC [16]. Thus, combining erdafitinib with immune checkpoint inhibitors is expected to enhance therapeutic efficacy. Although the frequency of MMR deficiency is not high, recognizing its characteristic clinicopathological features is crucial for optimizing UTUC treatment strategies. Although sequencing is required for a definitive LS diagnosis, identifying LS in MMR‐deficient patients offers important benefits, such as early screening for colorectal and other associated cancers, as well as proactive surveillance for blood relatives.

The prevalence of MMR deficiency differed between datasets (13% in this study vs. 4% in Fujii et al.), likely due to several factors. First, differences in assessment methods may contribute—our study used IHC to evaluate MMR protein expression, while Fujii et al. analyzed gene mutations, which may not yield identical results. Second, cohort differences such as age, tumor grade, and stage may influence the frequency of MMR deficiency. Nevertheless, MMR deficiency was consistently associated with favorable prognosis across datasets, indicating its potential clinical relevance in UTUC. Given its prognostic value, evaluating MMR deficiency in all UTUC patients may aid treatment stratification. Notably, it appears more frequent in younger patients and those with low NLR, suggesting that careful evaluation of MMR status may be particularly important in these cases.

MMR‐deficient tumors often exhibit abundant TILs, making immune cell infiltration in the tumor microenvironment (TME) a key factor in prognosis and treatment decisions. In this study, MMR‐deficient UTUC was associated with increased CD8‐positive TILs, which are known to predict improved prognosis in UTUC [17]. Indeed, a positive correlation between MMR deficiency and intratumoral CD8‐positive lymphocytes has been reported in various cancers [18]. MMR deficiency may lead to high TMB and MSI, increasing neoantigen expression and enhancing tumor immunogenicity, thereby attracting CD8‐positive T cells to the TME [19]. In MMR‐deficient colorectal cancers, CD8‐positive intraepithelial lymphocytes often cluster around crypts with MMR‐inactivating mutations, suggesting a role in tumor evolution [20]. Similar mechanisms may underlie the association in UTUC, though further studies are needed to clarify the molecular pathways involved. Interestingly, Cheng et al. further demonstrated a strong link between high stromal TILs and the PD‐1/PD‐L1/CD8 axis in UTUC [21]. Consistently, our study found an association between CD8‐positive and PD‐L1‐positive TILs. These findings suggest that CD8‐positive TIL density may serve as a useful predictor of MMR status, and their interaction with PD‐L1‐positive TILs could play a crucial role in treatment outcomes for MMR‐deficient UTUC. Additionally, MMR‐deficient UTUC showed higher TMB and is likely more immunogenic, suggesting potential responsiveness to PD‐1/PD‐L1 inhibitors. In colorectal cancers, MMR deficiency is an established predictive biomarker for immune checkpoint therapy [22]. However, further clinical studies are needed to clarify its therapeutic relevance in UTUC.

MMR plays an important role in the TME and immune response. In this study, GSEA revealed that MMR‐mutated UTUC is associated with MYC targets v1, OXPHOS, adipogenesis, and UPR. MYC is a key transcription factor involved in various cancer‐related pathways. Although the direct relationship between MMR and MYC remains unclear, MMR deficiencies disrupt genomic stability, which may influence MYC regulation. Additionally, the effects of MMR mutation on the TME, including OXPHOS, lipogenesis, and UPR, might play a pivotal role in determining UTUC malignant behavior. Notably, the relationship between MMR mutation and OXPHOS was previously reported. Cai et al. showed that a favorable prognosis in MSI gastric cancer is linked to OXPHOS‐related pathways, with increased apoptosis resulting from mitochondrial protein upregulation in OXPHOS, ROS pathways, and MYC targets [23]. Consistent with this, our GO analysis showed that genes upregulated in MMR‐mutated cases were enriched in mitochondria and inner mitochondrial membranes. Furthermore, recent studies indicate that OXPHOS downregulation is associated with poor clinical outcomes across cancers and correlates with a gene signature characteristic of invasive and metastatic tumors [24]. Another study reported that an activated oxidative metabolic state corresponds to a lower risk of progression in BLCA cell lines [25]. These findings suggest that the interaction between MMR mutations and OXPHOS genes may contribute to a favorable prognosis in UTUC. In contrast, the MMR‐normal group exhibited enrichment in malignant cell functions such as ECM, FA, and the PIK‐AKT signaling pathway. The interaction between ECM and FA is known to play a significant role in the TME, contributing to therapy resistance and tumor progression [26, 27]. FA is also involved in activating the PI3K‐AKT pathway, which is central to malignant behaviors [27]. Overall, these findings underscore the importance of MMR status as a prognostic indicator as it influences key biological processes that drive malignant behaviors of UTUC.

We also identified GALNT12 and FRMD3 as the predictive genes for MMR mutation in UTUC for the first time, to our knowledge. The discovery of these genes may open new avenues for further analysis in MMR‐mutated UTUC. GALNT12, a member of GALNT family members, mediates O‐glycosylation, a post‐translational modification of proteins [28]. Although its role in carcinogenesis has been reported in several studies, in prostate cancer, GALNT12 has been shown to suppress tumor cell proliferation, migration, invasion, cell division, and bone‐specific metastasis [28]. FRMD3 is a protein containing a FERM domain, which plays a structural role in linking the cell membrane to the cytoskeleton [29]. In several cancers, FRMD3 has been reported to inhibit tumor growth and metastasis, highlighting its potential role as a tumor suppressor gene [29]. Given their tumor‐suppressive roles, it is plausible that GALNT12 and FRMD3 contribute to the favorable prognosis observed in MMR‐mutated UTUC. Indeed, high FRMD3 expression was associated with favorable prognosis in UTUC, suggesting its potential as a marker for both MMR mutation detection and prognosis prediction.

This study had some limitations. First, its retrospective design necessitates validation through prospective studies. Second, cases with decreased MMR expression require confirmation of germline mutations in MSH2, MSH6, MLH1, and PMS2 through sequencing. Additionally, obtaining detailed family histories is essential to identify LS‐related tumors and their age of onset. Third, while our bioinformatics analysis revealed an association between MMR‐mutated UTUC, MYC, and OXPHOS genes, and the overexpression of GALNT12 and FRMD3, further detailed investigations are necessary. Although the bioinformatics analysis was performed using Fujii's dataset [2], future research should incorporate RNA‐seq analysis on our own samples for validation. Such studies could provide novel insights into the biology of MMR‐mutated UTUC. Fourth, the impact of MMR status on chemotherapy response in UTUC remains unclear. Although MMR‐deficient tumors may show reduced sensitivity to 5‐FU monotherapy, MSI‐H/dMMR colorectal cancers treated with IFL have shown improved five‐year disease‐free survival [30]. The hypermutated state from MMR deficiency may also influence treatment response through secondary mutations [30]. Further clinical trials are needed to clarify this relationship in UTUC.

In conclusion, this study revealed that MMR‐deficient UTUC was associated with younger age, lower NLR, less pathologically aggressive features with CD8‐positive TILs, and a favorable prognosis. The identification of MMR expression proteins is crucial for recognizing UTUCs with suspected MMR gene abnormalities, providing essential information for treatment strategies and surveillance.

## Author Contributions

**Go Kobayashi:** conceptualization, methodology, software, data curation, investigation, validation, formal analysis, visualization, project administration, writing – original draft. **Tetsutaro Hayashi:** writing – review and editing, conceptualization, methodology, data curation, investigation, validation, formal analysis, supervision, project administration, visualization. **Yohei Sekino:** writing – review and editing, methodology, project administration, data curation, supervision, funding acquisition, resources. **Kenichiro Ikeda:** writing – review and editing, visualization, investigation, formal analysis, data curation. **Hikaru Nakahara:** investigation, data curation, formal analysis, visualization, writing – review and editing. **Kohei Kobatake:** writing – review and editing. **Keisuke Goto:** writing – review and editing. **Daiki Taniyama:** writing – review and editing. **Kazuya Kuraoka:** writing – review and editing. **Shintaro Akabane:** writing – review and editing. **Hiroaki Niitsu:** writing – review and editing. **Takao Hinoi:** writing – review and editing. **Kazuhiro Sentani:** writing – review and editing, conceptualization, methodology, resources, funding acquisition. **Nobuyuki Hinata:** writing – review and editing.

## Ethics Statement

This retrospective study was approved by the Ethics Committee of Hiroshima University (authorization number: E20001‐9923) and was conducted in accordance with the Declaration of Helsinki of 1975.

## Consent

Informed consent was obtained from each patient.

## Conflicts of Interest

Go Kobayashi, Tetsutaro Hayashi, Yohei Sekino, Kenichiro Ikeda, Hikaru Nakahara, Kohei Kobatake, Keisuke Goto, Daiki Taniyama, Kazuya Kuraoka, Shintaro Akabane, Hiroaki Niitsu, Takao Hinoi, and Kazuhiro Sentani declare no conflicts of interest. Nobuyuki Hinata is an Editorial Board member of the International Journal of Urology and a coauthor of this article. To minimize bias, he was excluded from all editorial decision‐making processes related to the acceptance of this article for publication.

## Supporting information


**Figure S1.** The images depict positive control slides featuring colorectal cancer or gastric cancer with Lynch syndrome (MLH1/PMS2 loss).

**Figure S2.** Mismatch repair (MMR) mutation in TCGA BLCA datasets. (a) Kaplan–Meier plots illustrating overall survival (OS) of BLCA patients. (b) Proportion of MMR‐mutation versus MMR‐normal cases among surviving and deceased patients in the AACR GENIE BLCA study. Comparison of tumor mutation burden (TMB) between MMR‐mutated and MMR‐normal cases in (c) the TCGA BLCA study and (d) the AACR GENIE BLCA study. Statistical significance was assessed using the Mann–Whitney *U* test.

**Figure S3.** Relationship between GALNT12 and FRMD3 gene expression and oncological outcomes in the upper tract urothelial carcinoma (UTUC) study by Fujii et al. (a) Correlation analysis between GALNT12 and FRMD3 expression. *R* represents the correlation coefficient, and statistical significance was assessed using Spearman’s rank correlation test. (b, c) Comparison of GALNT12 and FRMD3 gene expression levels between surviving and deceased patients or between patients with and without disease progression. Statistical significance was assessed using the Mann–Whitney *U* test. Kaplan–Meier plots of (d, e) GALNT12 and (f, g) FRMD3 gene expression illustrating (d, f) cancer‐specific survival (CSS) and (e, g) progression‐free survival (PFS) in UTUC patients.

**Table S1.** Relationship between PD‐L1 and CD8 expression in UTUC.
**Table S2**. Results of Lasso regression analysis for predicting MMR mutation.

**Data S1.** The Python codes used in this study are presented.

**Data S2.** List of Supplementary References.

**Data S3.** Details of bioinformatics analysis are provided.
