# Supplementary figures and images for: Clinicopathological Characteristics of Upper Tract Urothelial Cancer With Loss of Immunohistochemical Expression of Mismatch Repair Proteins
Source: Int J Urol. 2025 Jun 9;32(9):1257–69. doi: 10.1111/iju.70146 (PMC12410129; doi:10.1111/iju.70146)

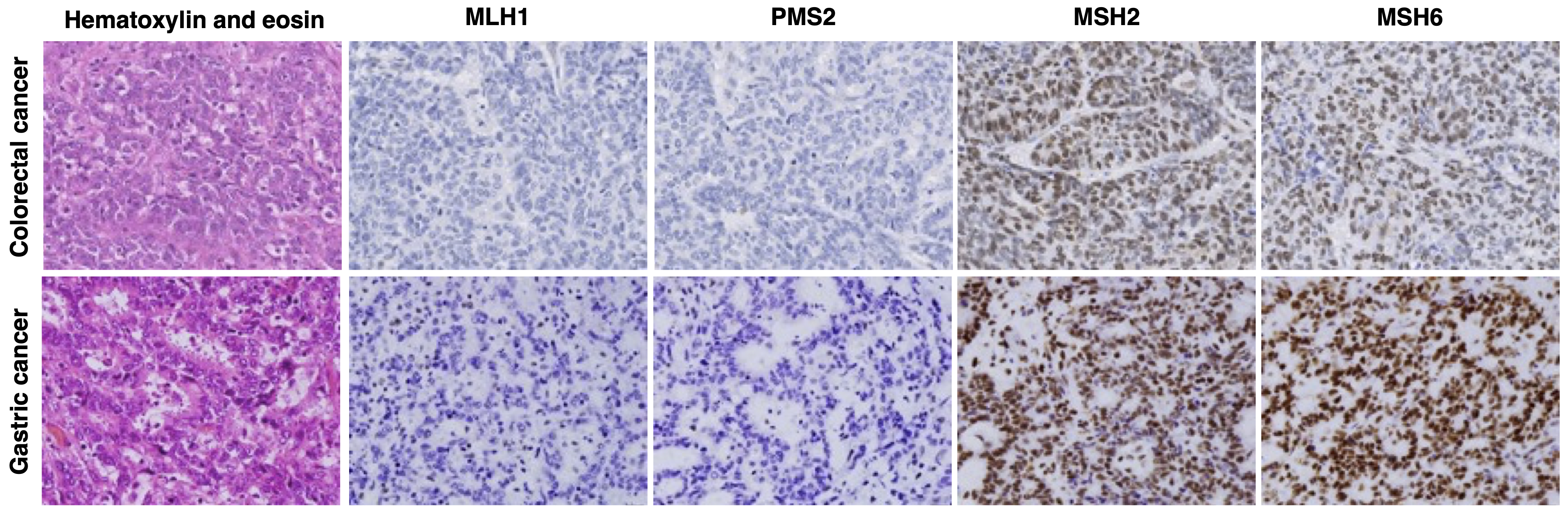

Supplement: Supplementary file 1 — Figure S1. The images depict positive control slides featuring colorectal cancer or gastric cancer with Lynch syndrome (MLH1/PMS2 loss). [file IJU-32-1257-s004.tiff]

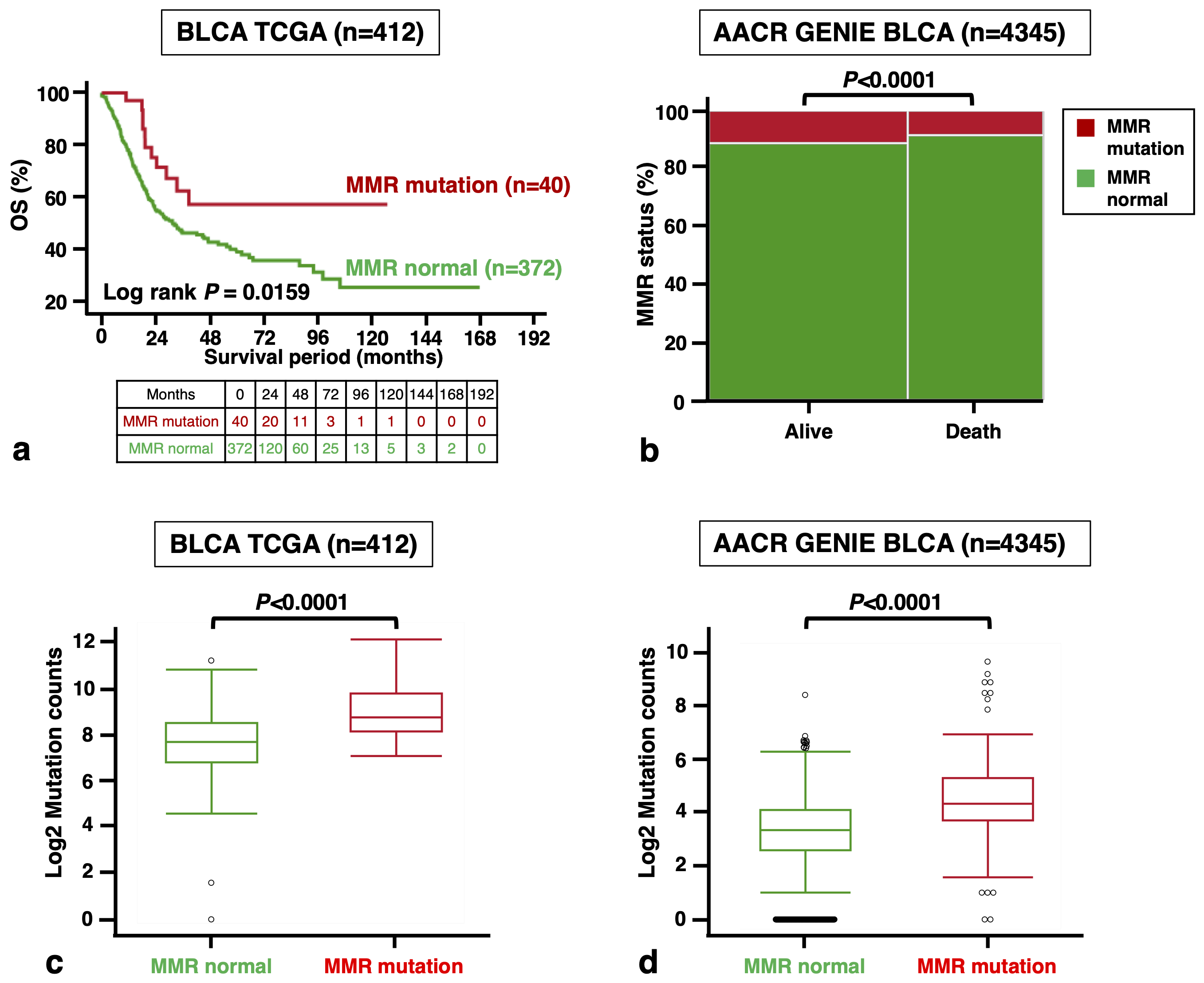

Supplement: Supplementary file 2 — Figure S2. Mismatch repair (MMR) mutation in TCGA BLCA datasets. (a) Kaplan–Meier plots illustrating overall survival (OS) of BLCA patients. (b) Proportion of MMR‐mutation versus MMR‐normal cases among surviving and deceased patients in the AACR GENIE BLCA study. Comparison of tumor mutation burden (TMB) between MMR‐mutated and MMR‐normal cases in (c) the TCGA BLCA study and (d) the AACR GENIE BLCA study. Statistical significance was assessed using the Mann–Whitney U test. [file IJU-32-1257-s002.tiff]

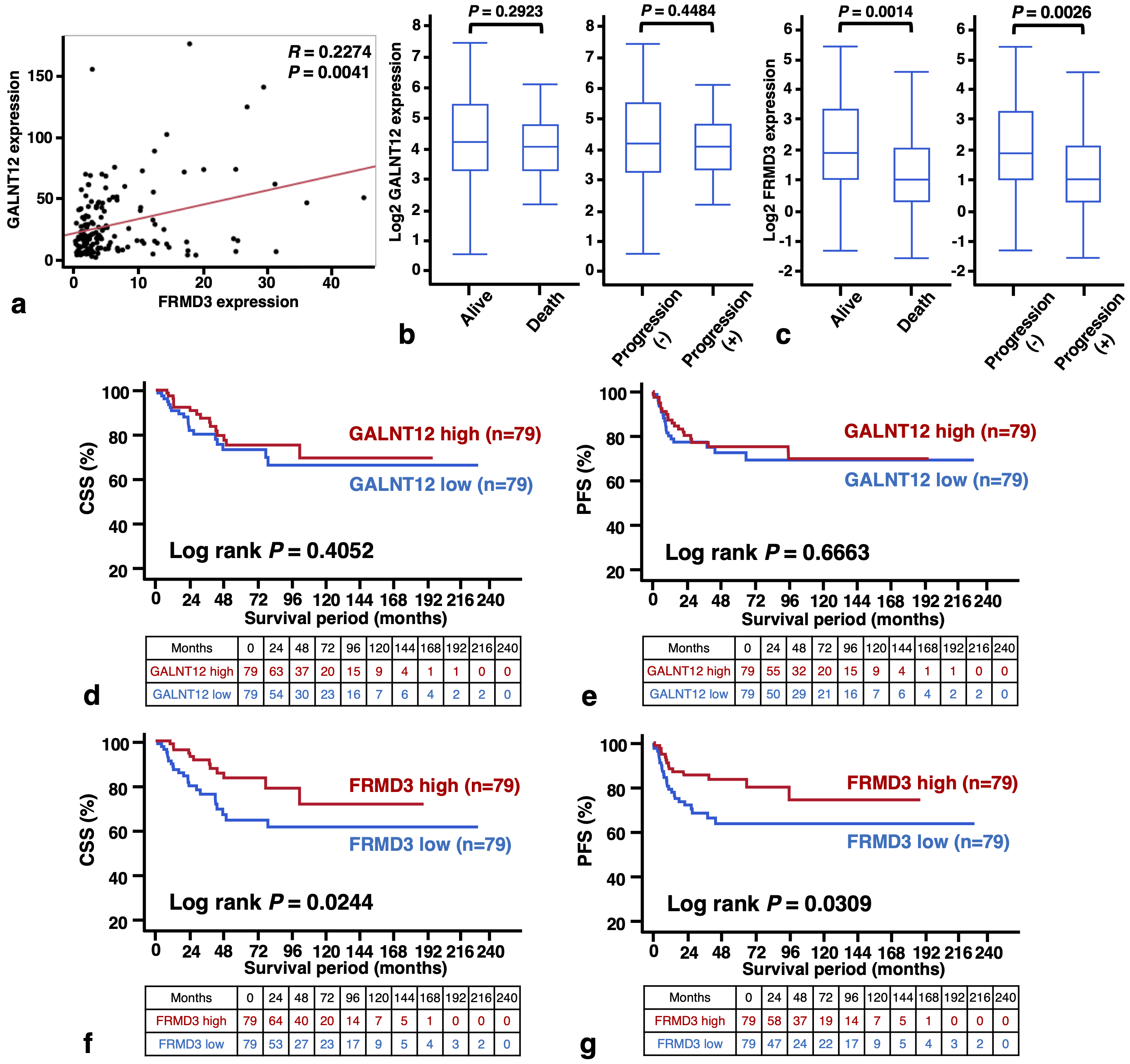

Supplement: Supplementary file 3 — Figure S3. Relationship between GALNT12 and FRMD3 gene expression and oncological outcomes in the upper tract urothelial carcinoma (UTUC) study by Fujii et al. (a) Correlation analysis between GALNT12 and FRMD3 expression. R represents the correlation coefficient, and statistical significance was assessed using Spearman’s rank correlation test. (b, c) Comparison of GALNT12 and FRMD3 gene expression levels between surviving and deceased patients or between patients with and without disease progression. Statistical significance was assessed using the Mann–Whitney U test. Kaplan–Meier plots of (d, e) GALNT12 and (f, g) FRMD3 gene expression illustrating (d, f) cancer‐specific survival (CSS) and (e, g) progression‐free survival (PFS) in UTUC patients. [file IJU-32-1257-s003.tiff]
