# Supplementary material for: Clinicopathological Characteristics of Upper Tract Urothelial Cancer With Loss of Immunohistochemical Expression of Mismatch Repair Proteins
Source: Int J Urol. 2025 Jun 9;32(9):1257–69. doi: 10.1111/iju.70146 (PMC12410129; doi:10.1111/iju.70146)
Supplement: Supplementary file 4 — Table S1. Relationship between PD‐L1 and CD8 expression in UTUC. Table S2. Results of Lasso regression analysis for predicting MMR mutation. [file IJU-32-1257-s005.docx]

**Supplementary Table 1 Relationship between PD-L1 and CD8 expression in UTUC**

|  |  | CD8 positive TILs (n=36) | CD8 negative TILs (n=82) | P-value |
| --- | --- | --- | --- | --- |
| PD-L1 in TCs | Positive | 7 (41%) | 10 (59%) | 0.3018 |
|  | Negative | 29 (29%) | 72 (71%) |  |
| **PD-L1 in TILs** | **Positive** | 16 (43%) | 21 (57%) | **0.0423** |
|  | Negative | 20 (25%) | 61 (75%) |  |
| Abbreviations: PD-L1, programmed death ligand 1; TCs, tumor cells; TILs, tumor-infiltrating lymphocyte.  P values were calculated with Fisher's exact test.  Bold values show the statistical significance. | | | | |

**Supplementary Table 2 Results of Lasso regression analysis for predicting MMR mutation**

| Gene | Standardized estimate | *P*-value |
| --- | --- | --- |
| **GALNT12** | 12.548913 | **<0.0001** |
| **FRMD3** | 7.8927435 | **0.0003** |
| RHOQP3 | 6.4665625 | 0.0517 |
| MGAT4B | 6.6825982 | 0.0680 |
| ANP32BP3 | 6.3649058 | 0.0691 |
| RP11-661A12.14 | 5.3336369 | 0.0970 |
| RADX | 3.7280801 | 0.2414 |
| COPB1 | 3.2428010 | 0.4167 |
| IPO7P2 | 2.1156742 | 0.5459 |
| POLR1B | 2.3396143 | 0.6117 |
| DLAT | 1.8239355 | 0.6707 |
| P values were calculated with Wald chi-squared tests.  Bold values show the statistical significance. | | |
