# Supplementary material for: Clinicopathological Characteristics of Upper Tract Urothelial Cancer With Loss of Immunohistochemical Expression of Mismatch Repair Proteins
Source: Int J Urol. 2025 Jun 9;32(9):1257–69. doi: 10.1111/iju.70146 (PMC12410129; doi:10.1111/iju.70146)
Supplement: Supplementary file 5 — Data S1. The Python codes used in this study are presented. [file IJU-32-1257-s001.docx]

1. **Python cord for volcano plot**

import pandas as pd

import numpy as np

from scipy import stats

import statsmodels.stats.multitest as smm

import matplotlib.pyplot as plt

# Load data

data = pd.read_csv('File name.csv', index_col=0)

# Classify samples based on the MMR row

mmr_values = data.loc['MMR']

mutated_samples = mmr_values[mmr_values == 1].index # Samples with MMR-mutated

normal_samples = mmr_values[mmr_values == 0].index # Samples with MMR-normal

# Create dataframes for MMR-mutated and MMR-normal

mutated_data = data[mutated_samples]

normal_data = data[normal_samples]

# Remove the MMR row

data = data.drop(index='MMR')

# List to store results

results = []

# Loop through each gene

for gene in data.index:

# Get expression data for each gene as Series

mutated_expression = mutated_data.loc[gene].squeeze().replace(0, np.nan).dropna().astype(float)

normal_expression = normal_data.loc[gene].squeeze().replace(0, np.nan).dropna().astype(float)

# Perform t-test if the number of samples is sufficient

if len(mutated_expression) > 1 and len(normal_expression) > 1:

t_stat, p_value = stats.ttest_ind(mutated_expression, normal_expression, equal_var=False)

log2_fc = np.log2(mutated_expression.mean() / normal_expression.mean())

results.append([gene, log2_fc, p_value])

# Convert results into a dataframe

results_df = pd.DataFrame(results, columns=['Gene', 'Log2_FC', 'P_Value'])

# Perform FDR correction (Benjamini-Hochberg method) to adjust p-values

alpha = 0.05

results_df['Adjusted_P_Value'] = smm.multipletests(results_df['P_Value'], alpha=alpha, method='fdr_bh')[1]

# Filter significant genes

significant_genes = results_df[

(results_df['Adjusted_P_Value'] < 0.05) &

(abs(results_df['Log2_FC']) >= np.log2(1.5))

]

# Filter genes highly expressed in MMR-mutated (positive Log2_FC)

mutated_high_genes = significant_genes[significant_genes['Log2_FC'] > 0]

# Filter genes highly expressed in MMR-normal (negative Log2_FC)

normal_high_genes = significant_genes[significant_genes['Log2_FC'] < 0]

# Save results to CSV

mutated_high_genes.to_csv('MMR_mutated_high_genes.csv', index=False)

normal_high_genes.to_csv('MMR_normal_high_genes.csv', index=False)

# Create volcano plot

plt.figure(figsize=(10, 7))

# Plot all genes in gray

plt.scatter(results_df['Log2_FC'], -np.log10(results_df['Adjusted_P_Value']), color='grey', alpha=0.5)

# Plot genes in MMR-mutated in red

plt.scatter(mutated_high_genes['Log2_FC'], -np.log10(mutated_high_genes['Adjusted_P_Value']), color='red', alpha=0.8, label='MMR-mutated')

# Plot genes in MMR-normal in blue

plt.scatter(normal_high_genes['Log2_FC'], -np.log10(normal_high_genes['Adjusted_P_Value']), color='blue', alpha=0.8, label='MMR-normal')

# Set axis labels

plt.xlabel('Log2 Fold Change', fontsize=13)

plt.ylabel('-log10(Adjusted P-Value)', fontsize=13)

plt.xticks(fontsize=12)

plt.yticks(fontsize=12)

# Adjust X and Y axis ranges

x_min, x_max = -8, 8

y_min, y_max = 0, 18

plt.xlim(x_min, x_max)

plt.ylim(y_min, y_max)

# Add threshold lines (optional)

plt.axhline(y=-np.log10(0.05), color='black', linestyle='--', label='P-Value = 0.05')

plt.axvline(x=np.log2(1.5), color='black', linestyle='--', label='Fold Change = 1.5')

plt.axvline(x=-np.log2(1.5), color='black', linestyle='--')

plt.legend()

plt.show()

1. **Waterfall plot**

import pandas as pd

import matplotlib.pyplot as plt

import seaborn as sns

# Read in the data

data = pd.read_csv(“File name.csv”, low_memory=False)

# Exclude missing values

data = data.dropna(subset=[“Tumor_Sample”, “MMR”, “Hugo_Symbol”, “mutation type”])

# Split the data into MMR-positive and MMR-negative groups

mmr_mutation = data[data[“MMR”] == “MMR Mutation”]

mmr_normal = data[data[“MMR”] == “MMR Normal”]

# Oncoplot creation function

def plot_oncoplot_with_bars(data, title):

# Organize samples and mutation types for each gene

mutation_matrix = data.groupby([“Hugo_Symbol”, “Tumor_Sample”, “mutation type”]).size().reset_index(name=“Count”)

# Calculate the sample frequency (number of samples in which the mutation was detected) for each gene and extract the top 20

top_genes = (

mutation_matrix.groupby(“Hugo_Symbol”)[“Tumor_Sample”]

.nunique()

.sort_values(ascending=False)

.head(20)

.index

)

mutation_matrix = mutation_matrix[mutation_matrix[“Hugo_Symbol”].isin(top_genes)]

# Create a pivot table to prepare data for plotting

pivot_table = mutation_matrix.pivot_table(

index=“Hugo_Symbol”, columns=“Tumor_Sample”, values=“mutation type”, aggfunc="first”

)

# Calculate mutation counts (per sample) and gene frequencies (per gene)

sample_mutation_counts = pivot_table.notna().sum(axis=0)

gene_mutation_counts = pivot_table.notna().sum(axis=1)

# Sort the genes in order of gene frequency (highest to lowest)

sorted_genes = gene_mutation_counts.sort_values(ascending=False).index

pivot_table = pivot_table.loc[sorted_genes]

gene_mutation_counts = gene_mutation_counts.loc[sorted_genes]

# Assign colors to each category

mutation_types = mutation_matrix[“mutation type”].dropna().unique()

color_palette = sns.color_palette(“tab10”, len(mutation_types))

color_map = {

“Missense_Mutation“: ‘#1f77b4’, # blue

“Nonsense_Mutation”: “#ff7f0e”, # orange

“Frame_Shift_Del“: ‘#e377c2’, # pink

“Frame_Shift_Ins”: “#d62728”, # red

“In_Frame_Del“: ‘#9467bd’, # purple

“In_Frame_Ins”: “#8c564b”, # brown

“Silent“: ‘#7f7f7f’, # gray

“Splice_Site”: “#2ca02c”, # green

“Nonstop_Mutation“: ‘#bcbd22’, # yellow-green

“Translation_Start_Site”: “#17becf”, # light blue

}

# Create a color map

tile_colors = pivot_table.applymap(lambda x: color_map.get(x, “white”))

# Create a graph

fig = plt.figure(figsize=(16, 10))

# Get the number of genes and samples

num_genes = len(pivot_table.index)

num_samples = len(pivot_table.columns)

# Set grid spec (adjust width and height according to number of genes and samples)

gs = fig.add_gridspec(

3, 2,

width_ratios=[1, 0.3], # Modify width ratio

height_ratios=[1, len(pivot_table.index), 0.5],

hspace=0.17, wspace=0.17

)

# Mutation Count (top bar chart)

ax_bar_top = fig.add_subplot(gs[0, 0])

ax_bar_top.bar(range(num_samples), sample_mutation_counts.values, color=“gray”, align=“center”, width=1.0)

ax_bar_top.set_xlim(-0.5, num_samples - 0.5) # Match the width of the tile

ax_bar_top.set_ylabel(“Mutation Count”, fontsize=8)

ax_bar_top.set_ylim(0, 20) # Fix the maximum value of the Y-axis

ax_bar_top.set_xticks([])

ax_bar_top.spines[“right”].set_visible(False)

ax_bar_top.spines[“top”].set_visible(False)

# Gene Frequency (right-hand bar graph)

ax_bar_right = fig.add_subplot(gs[1, 1])

ax_bar_right.barh(range(num_genes), gene_mutation_counts.values, color=“gray”, align=“center”, height=1.0)

ax_bar_right.set_ylim(-0.5, num_genes - 0.5) # Match the height of the tile

ax_bar_right.set_xlabel(“Gene Frequency”, fontsize=10)

ax_bar_right.set_yticks(range(num_genes))

ax_bar_right.set_yticklabels(gene_mutation_counts.index, fontsize=10)

ax_bar_right.invert_yaxis() # Invert to order from high frequency

ax_bar_right.spines[“right”].set_visible(False)

ax_bar_right.spines[“top”].set_visible(False)

# Tile plot (main plot)

ax_main = fig.add_subplot(gs[1, 0])

for i, row in enumerate(tile_colors.iterrows()):

for j, color in enumerate(row[1]):

ax_main.add_patch(plt.Rectangle((j, i), 1, 1, color=color)) # Draw the tiles

ax_main.set_xlim(0, len(pivot_table.columns))

ax_main.set_ylim(0, len(pivot_table.index))

ax_main.set_xticks([x + 0.5 for x in range(len(pivot_table.columns))]) # Align to the center of the tile

ax_main.set_xticklabels([])

ax_main.set_yticks([y + 0.5 for y in range(len(pivot_table.index))]) # Align with the center of the tile

ax_main.set_yticklabels(pivot_table.index, fontsize=10)

ax_main.invert_yaxis() # Arrange the genes from top to bottom

# Create the legend

ax_legend = fig.add_subplot(gs[2, 0])

legend_elements = [

plt.Line2D([0], [0], color=color, lw=4, label=mutation_type)

for mutation_type, color in color_map.items()

]

ax_legend.legend(

handles=legend_elements, loc=“center”, ncol=5, fontsize=10, frameon=False, bbox_to_anchor=(0.5, -0.2)

)

ax_legend.axis(“off”) # Hide the axis

# Figure title and overall adjustment

fig.suptitle(title, fontsize=16, y=0.95)

plt.tight_layout()

plt.show()

# Plot for MMR Mutation group

plot_oncoplot_with_bars(mmr_mutation, “Oncoplot with Bars: MMR Mutation Group”)

# Plot for MMR Normal group

plot_oncoplot_with_bars(mmr_normal, “Oncoplot with Bars: MMR Normal Group”)
