# Supplementary material for: Clinicopathological Characteristics of Upper Tract Urothelial Cancer With Loss of Immunohistochemical Expression of Mismatch Repair Proteins
Source: Int J Urol. 2025 Jun 9;32(9):1257–69. doi: 10.1111/iju.70146 (PMC12410129; doi:10.1111/iju.70146)
Supplement: Supplementary file 6 — Data S2. List of Supplementary References. [file IJU-32-1257-s007.docx]

**List of Supplementary References**

[1] Kim K, Hu W, Audenet F et al. Modeling biological and genetic diversity in upper tract urothelial carcinoma with patient derived xenografts. *Nat Commun*. 2020; **11**: 1975.

[2] Robertson AG, Kim J, Al-Ahmadie H et al. Comprehensive Molecular Characterization of Muscle-Invasive Bladder Cancer. *Cell*. 2017; **171**: 540-56.e25.

[3] Sfakianos JP, Cha EK, Iyer G et al. Genomic Characterization of Upper Tract Urothelial Carcinoma. *Eur Urol*. 2015; **68**: 970-7.

[4] de Bruijn I, Kundra R, Mastrogiacomo B et al. Analysis and Visualization of Longitudinal Genomic and Clinical Data from the AACR Project GENIE Biopharma Collaborative in cBioPortal. *Cancer Res*. 2023; **83**: 3861-7.
