# Supplementary material for: Clinicopathological Characteristics of Upper Tract Urothelial Cancer With Loss of Immunohistochemical Expression of Mismatch Repair Proteins
Source: Int J Urol. 2025 Jun 9;32(9):1257–69. doi: 10.1111/iju.70146 (PMC12410129; doi:10.1111/iju.70146)
Supplement: Supplementary file 7 — Data S3. Details of bioinformatics analysis are provided. [file IJU-32-1257-s006.docx]

**Supplementary text**

**Gene set enrichment analysis (GSEA)**

GSEA software was downloaded from https://www.gsea-msigdb.org, and the samples were divided into MMR mutation and MMR normal groups. The gene sets of “h.all.v2024.1.\\hs.symbols.gmt” from the Molecular Signatures Database were downloaded. The number of permutations was set at 1000. Normalized enrichment scores, the nominal *P* value (NOM *P* value), and the false discovery rate (FDR) Q value were acquired. NOM *P* value <0.05 and FDR Q value <0.25 were considered to indicate significant enrichment.

**Screening of differentially expressed genes (DEGs)**

According to the RNA-seq database in the Fujii et al. study^(2)^, we divided genes into the MMR-mutated group and MMR-normal group, and the DEGs were used for screening by volcano plot. The threshold was 1.5-fold change and adjusted *P* <0.05. The volcano map of DEGs was constructed by installing Python 3.0 on Jupyter Notebook (version 6.3.0). The codes used are presented in Supplementary Data 1^(18)^**.**

**Gene ontology and functional enrichment**

To determine the biological function of MMR mutation and its related DEGs, we annotated them with Gene Ontology (GO) and the Kyoto Encyclopedia of Genes and Genomes (KEGG), using DAVID v6.8 (<https://david.ncifcrf.gov/>) and ShinyGO 0.77 (<http://bioinformatics.sdstate.edu/go77/>). The results of the GO analysis were categorized into Biological Process (BP), Molecular Functions (MF), and Cellular Component (CC). Results with FDR <0.05 were considered statistically significant.
